# Cellular Network Radio Monitoring and Management through Virtual UE Probes: A Study Case Based on Crowded Events

**DOI:** 10.3390/s21103404

**Published:** 2021-05-13

**Authors:** Eduardo Baena, Sergio Fortes, Özgü Alay, Min Xie, Håkon Lønsethagen, Raquel Barco

**Affiliations:** 1 Instituto de Telecomunicación (TELMA), Universidad de Málaga, CEI Andalucía TECH, E.T.S. Ingeniería de Telecomunicación, Bulevar Louis Pasteur 35, 29010 Málaga, Spain; sfr@ic.uma.es (S.F.); rbm@ic.uma.es (R.B.); 2 Simula Metropolitan Center for Digital Engineering, Pilestredet 52, 0167 Oslo, Norway; ozgu@simula.no; 3 Telenor Research, Telenor, Snarøyveien 30, 1360 Fornebu, Norway; min.xie@telenor.com (M.X.); hakon.lonsethagen@telenor.com (H.L.)

**Keywords:** cellular performance, operators, measurements, probe, crowds

## Abstract

Although log processing of network equipment is a common technique in cellular network management, several factors make said task challenging, especially during mass attendance events. The present paper assesses classic methods for cellular network measurement and acquisition, showing how the use of on-the-field user probes can provide relevant capabilities to the analysis of cellular network performance. Therefore, a framework for the deployment of this kind of system is proposed here based on the development of a new hardware virtualization platform with radio frequency capabilities. Accordingly, an analysis of the characteristics and requirements for the use of virtual probes was performed. Moreover, the impact that social events (e.g., sports matches) may have on the service provision was evaluated through a real cellular scenario. For this purpose, a long-term measurement study during crowded events (i.e., football matches) in a stadium has been conducted, and the performances of different services and operators under realistic settings has been evaluated. As a result, several considerations are presented that can be used for better management of future networks.

## 1. Introduction

Modern mobile network management systems have undergone numerous developments and advances in recent years. The heterogeneity and the ever-increasing flexibility of the resources delivered in 5G networks, driven by the virtualization of network functions (NFV) [1] allow integrating big data tools into operational support systems (OSS) under the paradigm of the software-defined networks (SDN) orchestration. Thus, the functions of self organizing networks (SON) [2] that intended to automate many of the tasks performed by network engineers are becoming increasingly plausible. Despite all these innovations, some problems persist and make the management of cellular networks a complicated task. This fact is evidenced by incidents that cellular network monitoring systems may not notice. That is the case for sleeping cells, i.e., areas that, either due to interference, low coverage or any other radio problem, cause a low quality of experience (QoE) for the user. Such unnoticed incidents are especially significant in places where events with large concentrations of people (i.e., crowds) are held. Besides, troubleshooting issues in such locations can be challenging without a user terminal identifying the root causes and when using exclusively network performance indicator traces. Although it is known from field experience that the inclusion of user traces is very beneficial [3] for any task of the network operations, administration and maintenance (OAM), it has not yet been possible to make a systematic compilation to serve as a regular input for the SON system. In this context, drive tests carried out to map network deployments represent the state of the network in a specific time framework and do not capture the network evolution. Although minimization of drive test techniques (MDT) [4] can reduce the high cost of these tests, it is still very expensive to have periodic monitoring that reflects the conditions at any given moment. On the other hand, operators prefer probing methods that prevent the active intervention of the users.

The technological advancement of development hardware based on general-purpose platforms (GPPs) and software defined radio (SDR) makes possible the integration of flexible network elements that can be managed and configured by means of container-based virtualization applications such as Docker [5]. This availability is becoming a trend towards the use of open-source base stations replacing small cells that could be deployed by users and integrated into the 5G network [6,7]. With this approach, it would also be possible to enable these GPPs and SDR platforms as virtualized User Equipment (UE) probes (or vUE probes) to assist in network management, especially in conflicting places in a city, such as crowded events [8,9]. Thus, the advantages of this functionality are multiple: from testing new standards and configurations or assisting in troubleshooting to regular logging for traffic/usage profiling purposes, among many others. Significantly, the scenarios involving crowds lack traceability at the locations where they appear.

In this regard, the contributions of this work include:

1. A novel framework for the inclusion of user side trace information based on virtual UE probes (vUE probes) in OAM systems.
2. A case study based on crowded events illustrating the capabilities of these probes by characterizing their cross-layer/multi-operator acquisition of data. The combination of radio network layer information and different user application-level measurements goes beyond the previous studies.
3. A novel assessment of a crowded scenario (Stadium) based on a long-term measurement campaign, including the deployment of several probes connected to different operators, provides innovative insighte into how social events impact the network (before, during and after an event). From this case, the shortcomings, challenges and further applications of the proposed framework and the use of vUE probes were identified.

This paper is divided as follows: In Section 2, the related works and the classical approach of SON management are presented. In Section 3, the presentation of the framework of the use of vUE probing and its possible applications are discussed. Section 4 details a real crowded scenario (i.e., a football stadium) where the proposed framework was applied, thereby characterizing the capabilities of the approach and showing the insights into the impact of such events from a cross-layer perspective. From this, Section 5 focuses on the identification of the applications, challenges and ways forward for future vUE probe systems. Finally, Section 6 presents the conclusions of this work.

## 2. Related Work

While the support of novel technologies, such as the SDR and GPP platforms mentioned above, would make vUE probes possible, the current literature has not yet explored this topic in depth. However, plenty of previous works on the general concept of network monitoring are associated with the present work. In this way, the use of UE traces is a resource used very frequently, and numerous sources use it in network management literature. However, no systematization of its collection is yet specified, and multiple purposes in different campaigns are explained. This fact confirms the assumption that regularly obtained user data are valuable inputs. The following summarized articles are several examples of its many applications.

The work in [10] presents the design of a new measurement tool for Android capable of working with multiple radio technologies: 3G, WiFi (wireless fidelity) and LTE (long term evolution). The results analyzed include the round trip time (RTT), power consumption, throughput and delay under different circumstances and taking into account the different UE states.

The paper [11] also used monitoring applications at the UEs for quantifying network quality parameters, such as data rate, web page loading speed, voice quality and network coverage. However, no further assessment of the gathered data was provided. Similarly, the paper [12] focused on the comparison of the accuracy of UEs and sniffers for measuring the data rates of LTE and WiFi networks in burst or isolated transmission conditions. Based on these results, the work proposed an environmental model with some software alternatives for providing these measurement capabilities on mobile phones and base stations.

Likewise, the primary purpose in [13] was to improve the visibility and understanding of the complex mobile network performance. Differences across carriers, access technologies, geographic regions and over time were assessed. The associated details of mobile networks were very heterogeneous, making it challenging to detect service degradations properly. Additional parameters such as RTT, throughput or DNS resolutions and traceroutes were studied, allowing them to identify reasons behind some persistent problems. Despite the relevant information collected, the need for more monitoring and diagnosis to improve network performance was noted.

Other machine learning (ML) techniques were explored to map key performance indicators (KPI) to key quality indicators (KQIs) in [14]. From a campaign of measurements from the user terminal in a live LTE network, predictive modeling of FTP was performed and later extended to a video service [15]. In a similar way [16], an ML system was proposed to analyze the enormous amounts of data generated to monitor network performance. The work is considered preliminary, emphasizing the challenges and importance of latency prediction on operational mobile networks.

Besides, as mentioned in the introduction, virtualized probes as a tool for network monitoring and management as a framework are also scarce. In this way, different approaches have been proposed: In [17] the authors described the need to implement probes based on mobile edge computing technology for QoE monitoring without descriptively going into implementation details. Likewise, the contribution [11] describes the use of UE probes that are compiled in a crowdsourced manner. Although it proposes a possible architecture for their collection and uses in network management, its implementation can be difficult and costly and requires the users’ collaboration, which is not always possible.

The work in [18] introduced ORCA: An “operator classifier” for identifying patterns and disclosing exclusive aspects of mobile network operators in noisy crowdsourced datasets. That study was focused on a set of parameters commonly found on such datasets, contrary to the rest of related works that focused only on one or very few parameters. The gathered data, fundamentally the latency, allowed them to identify of different operators, although using all available features increased the accuracy of the classifier. In addition, the quality of experience of web services has been experimentally measured with probes by collecting user traces, some examples being [19,20].

Moreover, during the last few years, there has been a growing interest in developing tools that allow the capture of packets from the radio layers to deepen the influence of the configurations at this level. In [21], a software tool that allows access to the scheduling information by decoding the LTE control channel, OWL, was introduced. This work showed a one-day data collection campaign: radio network temporary identifier (RNTI) data-rates and MCS (modulation and coding scheme) assignments were shown.

Later, Ref [22] presented a complete open source solution (Mobil Insight) capable of obtaining messages from the cellular network protocol stack capable of being installed on any mobile commercial-off-the-shelf (COTS) platform. This software was tested in the present work as a complement to the virtual probe. Other commercial tools available on the market offer similar features to those mentioned above, although they are not open source [23,24].

It is also worth noting that the aforementioned softwarized environments are giving rise to a renewed interest in benchmarking the possible scenarios that will take place in 5G networks, such as those shown in [25]. This work demonstrates at a computational level the effects of some of the IoT protocols.

In terms of social crowds/events analysis, which will focus on the application of vUE probes in the present study, the studies done so far on the impact on the cellular network are limited. The work in [26] focused on optimizing the performance of networks during crowded events. Here, Transport Control Protocol (TCP) metrics, such as round trip time (RTT) and packet loss, are inferred to evaluate network performance. Based on this data, features like dropped calls are reduced to improve Internet user throughput. In this way, two possible solutions for mitigating those problems were proposed: on one hand, radio network parameter tuning and opportunistic connection sharing, and on the other, a selected set of UEs could act as WiFi hotspots for other UEs in their vicinity, thereby reducing the already occupied radio channels.

A similar work presented in [27], studied the use of contextual information [28,29] for cellular network management in social events. Furthermore, a framework for using such contextual information that could be applied to the vUE use case was described. However, unlike the present work, it was not supported by an extensive measurement campaign.

## 3. Framework

Since the management systems of 5G and next-generation mobile networks are likely to be user-centric [30], it will be necessary to include mechanisms capable of capturing and processing data from the user plane complementing network side log collection. Additionally, given the future network layers, split architectures will make this analysis more necessary [31].

Although the measurement of user data has been carried out in the scientific literature with applications to network management [18,32,33], there is still no consensus in the form of a standardized, unified proposal for its integration on OSS. Thus, although such integration has so far not been possible on a large scale in real deployments, the challenge of its complex management and maintenance rendered it expensive and unfeasible. The advent of virtualization, however, has led to a rethinking that could allow operators to manage and assimilate the virtual probes as part of the cellular network management systems [17].

**Figure 1:**
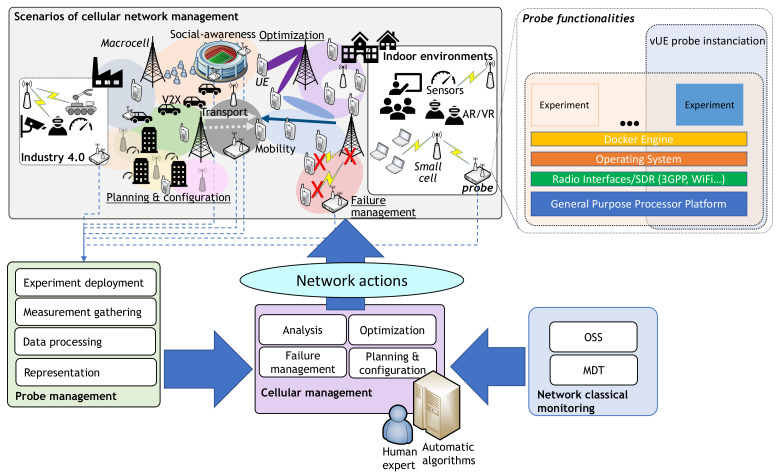
A high level diagram of the vUE probe framework.

In this sense, Figure 1 presents an overview of cell management in which vUE probing would be applied. Starting from the classical approach that would combine human expertise with automatic management (in the center of the scheme), monitoring performance indicators are derived from specific actions on the network. These actions can have various purposes ranging from fault management to optimization, and are applied according to multiple scenarios (top). In this specific management, direct information management of the probes comes into play, which in turn must also be managed (bottom left). Finally, the probes themselves can be instantiated to obtain scheduled or real-time information on the performance of the services or the radio environment (top right). The following subsections explain the architecture required to integrate both the vUE probes and the information contained therein for automatic cellular management.

### 3.1. Architecture

This section presents and details the proposed architecture for the use of the vUE probes. First of all, it assumes the complete virtualization of all network functions and separation of the data and user planes. In this way, the physical infrastructure (in the form of several GPPs and base band units or BBUs) can be mapped according to a slicing model of dynamic resource reservation on demand from the network manager [34].

#### 3.1.1. vUE Probes

Open radio access network (RAN) technologies will allow the resources to be converted into base stations or user terminals. The first step is to create containers with the execution of base stations or UEs. For this, software implementations of such elements are available, making use of the SDR platform where all the RF and PHY-related functions take place [35,36]. In this way, through configuration files, different virtual interfaces where the instantiated entity must connect (i.e., core functions [37]; IP address in the case of gNodeB) are set and all the necessary parameters (Mobile Country Code (MCC), Mobile Network Code (MNC), access point name (APN), eSIM, encryption type, frequencies, etc.) in the case of a UE. Therefore, the deployment of vUE probes is performed by instantiating the necessary hardware resources (including signal and computing processing units) selected to take the measurements with a proper parameter configuration to connect to the RAN.

Once established and tested, the operation of the vUE, including the connection to the container-based management system, can launch isolated virtualized scripts to take samples as needed.

Therefore, it is necessary to develop scripts to run on the vUEs and schedule them accordingly or on-demand, depending on whether a specific situation is found where the operator wants to troubleshoot more deeply from a UE perspective. Additionally, the inclusion of virtualization software in the probes (such as Docker [5]) allows instances to use the different radio functions to connect to different operators. Similarly, the use of a sniffer of the radio interface of the user could be instantiated to obtain a detailed capture of the signaling exchanged with the base station (using some of the above-mentioned open source tools such as Mobile Insight [22]).

#### 3.1.2. Experiments and Measurements

Several services can be launched as test scripts or experiments, depending on the application that needs to be dived into. Then, the vUE logs will have to be attached and timestamps matched and merged. In Table 1 the most relevant KQIs (describing quality of an end-to-end service in parameters that an end-user may directly experience) for various types of services (File Transfer Protocol (FTP), web (HyperText Transfer Protocol or HTTP), NetTest speed test [38] and video streaming) are shown. Each experiment could collect several of these services and indicators, or parts of them, depending on the relevant needs.

Such samples could optionally be labeled based on learning algorithms and based on previous samples. However this is not necessary in the first instance and could be used offline in both supervised and unsupervised learning algorithms.

In this way, the Web KQI parameters are defined as follows:

- First Paint: Time since the browser starts to render the page and shows the first hint of content on the screen. This is a good front-end benchmark of when a page appears to be starting to load.
- Page Load Time: Time it takes for the entire content of a web page to be downloaded and displayed in the browser.
- Fully Loaded Time: Time until there is 1.5 s of network inactivity after on-load, waiting up to a maximum of 5 s.

Regarding the FTP, the main KQI definitions are the following:

- Setup Time: denotes the time period needed to establish a connection to the FTP server, from sending the initial query to a server until the first data packet is received.
- Speed download: capacity to fully transmit a file of a given size per time unit.

Finally the ping service would have a single KQI:

- RTT: round trip time (RTT) or round trip latency, or simply response time, is the time from the sending of an ICMP packet to it being received again.

With respect with radio environment parameters, the most important parameters that could be obtained from a MODEM log are summarized in Table 2. Among these, the highlighted parameters are defined as follows.

- RSRP is the power of a reference received signal at the userside. Its value range is between −140 and −40 dBm approximately.
- RSRQ is a quality metric of the radio channel that is a ratio of RSRP and the total received signal power (as the sum of interference and noise) ranging between −19.5 and −3 dB.

**Table 2:** MODEM log parameters.

MODEM Log	
Identifiers	Integrated Circuit Card Identifier (ICCID) Physical Cell Identifier (PCI) Location Area Code (LAC) Cell ID (CID) eNodeB ID
Radio Parameters	Frequency Band Received Signal Code Power (RSCP) Reference Signal Received Power (RSRP) Reference Signal Received Quality (RSRQ) Received Signal Strength Indicator (RSSI) ratio of Signal to Interference (ECIO)

The experiments can be scheduled according to specific purposes (for instance, to reproduce a logging error at a given base station or to check for a sudden drop in performance at a specified time) or periodically according to a survey plan. Hence, these configurations can be set depending on the specific needs for the purposes intended.

Then the corresponding logs are sent by Secure SHell (SSH) to a Structured Query Language (SQL) database where they can be aggregated or examined online. From a control plane perspective, the data collected from the vUE probes can be gathered for several purposes, such as detecting anomalies and enhancing the channel selection process, learning about users behavior (mobility patterns, connection time, data usage, etc.) and extracting context information (number of users, number of base stations among others). Accordingly, concerning the user plane, data collected from vUE experiment campaigns could be incorporated to improve the service. In this way, supervised learning has been proposed for the QoE correlation model [15].

## 4. Assessment

In order to assess its capabilities with the current available technologies, the proposed framework has been implemented in a real scenario characterized by the appearance of huge crowds associated with sport events (football matches). Although only the programming, start-up and periodic data collection function were tested, with subsequent analysis of the data, it was not possible to act on the operator’s network, since they were carried out on a real network. Thus, it has not been possible to test the corrective actions on the network derived from the data collected.

### 4.1. Setup

The technology that has been used for implementation and the crowded events measurement campaign is from the open experimentation platform MONROE (Measuring Mobile Broadband Networks in Europe) [39]. The MONROE platform is a software environment created from an H2020 project that manages more than 300 virtualized probes distributed across Europe. It offers an isolated environment for running applications by wrapping the software in a complete file system containing everything needed for execution (i.e., virtual containers). In this way, containers share the underlying host resources but only include what they need to run applications. It is, therefore, possible to schedule multiple experiments and measurements to run on a probe to compare the performances of different mobile networks.

**Figure 2:**
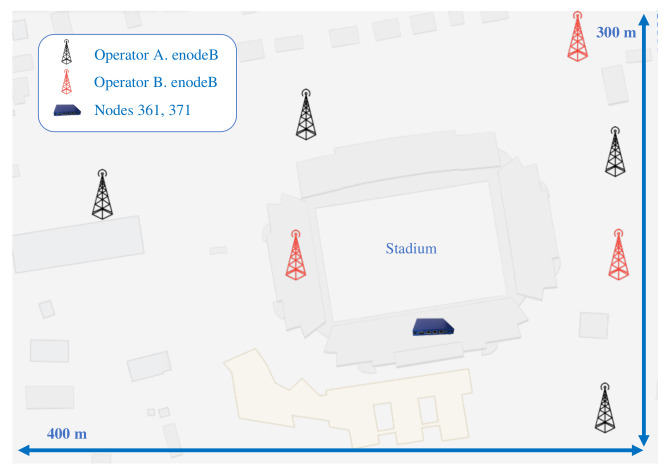
Approximate locations [41] of network elements in the stadium scenario.

The measurement campaigns have been executed by programming two probes equipped with LTE Cat6 radio interfaces and integrated with a container-based virtualization system developed by MONROE consortium [40]. These probes or nodes have been placed in Lerkendal Stadium of the Rosenborg BK team, located in Trondheim (Norway). In this area, two different operators A and B, have their base stations placed around the stadium, which enables the study of the influence of crowded events on the user-perceived service quality. In Figure 2, the approximate distribution of the base stations is shown according to a crowdsourced database online [41].

Beyond assessing the capabilities of the proposed framework, the research aimed to answer in this specific scenario whether the development of an event affects the quality of the service, and if it does, to determine the most influential factors and to which extent they have an impact. Additionally, it was of interest to find behavioral differences between operators.

### 4.2. Measurements Acquisition and Characterization

In order to carry out a study that was as detailed as possible on the impact of public attendance on the service performance, measurements at different layers were gathered. As described in Section 3.1.2, in the scenario, two probes were deployed at the same site within the data-center of the stadium where LTE coverage exists. The probes were node 371, equipped with two radio interfaces connected to both operator A and operator B, and node 361 with one interface connected to operator B.

This deployment was carried out in such a way that, despite being physically in the same place, two different average values of the signal received from operator B could be used for comparison. Furthermore, the existing hardware resources in both nodes were identical in terms of RAM (4 GB), Ethernet connection for management (1000 MBps) and available hard disk (16 GB), terebys emulating the same configuration (instantiation in the case of vUE probes). These settings emulate two possibilities of use under the same resources.

In addition to the MODEM parameters described in the previous section, an open-source traffic sniffer Mobile Insight [22] was used to incorporate the signalization packets from all LTE radio layers: Packet Data Convergence Protocol (PDCP), Network Access Stratum (NAS), Radio Resource Management (RRM), Radio Resource Control (RRC), Media Access Control (MAC) and physical (PHY). Based on these traces, radio layer indicators were extracted to help deepen the understanding of the influences on service performance and cross-layer effects.

Two measurement campaigns have been conducted over 16 months between August 2018 and December 2019, capturing the event days. oporator 1 recorded the impact of the Web service in 12 soccer matches every hour from 10:00 to 00:00 jointly with full-day MODEM Logs. In campaign 2, the impacts on FTP, NetTest and ping services (jointly with MODEM and Mobile Insight Logs) of 6 matches were captured with a finer granularity (15 min) from 2 h before and until 2 h after. The Table 3 shows the average number of samples captured per node.

### 4.3. Crowd Impact Analysis

Once the data were collected in both campaigns, analysis was required in order to showcase the capabilities of the proposed approach. Additionally, additional insights into the cross-layer and multi-operator nature of the impact of crowd-events are provided. In this way, an evaluation of the data previously described is shown from a top-down approach:

1. The temporal behavior is described from the point of view of the service KQIs.
2. The radio environment is analyzed based on the information obtained from the MODEM logs. A vision of the evolution of the physical signals can be obtained at a cell level.
3. A more specific analysis of all the radio communication with a much higher level of granularity and detail is given by processing the Mobile Insight traces.
4. The application of the multi-layer gathering of data is showcased based on the analysis of the correlations between the parameters of the radio layers and the service performance indicators, identifying the most relevant parameters for event management.

### 4.4. Service KQIs

**Figure 3:**
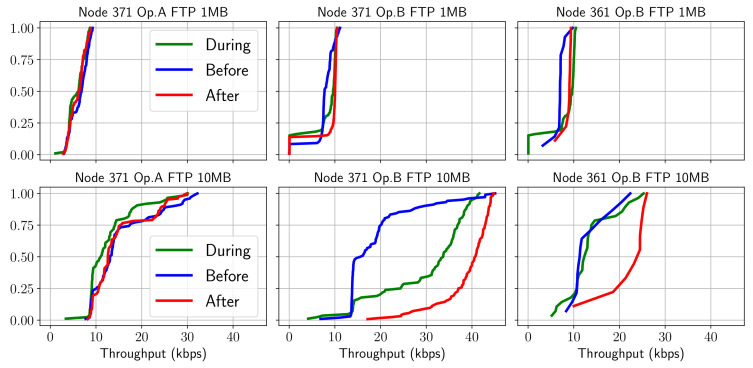
CDF of FTP throughput behavior before/during/after the match.

Firstly, an evaluation of the performance parameters (i.e., KQIs) for each of the services tested in the experiments has been carried out. The purpose was to test whether there was a substantial difference in behavior or characterization of behavior before/during/after each event. To this end, experimental cumulative probability distribution functions (CDF) have been obtained by filtering two hours before, during the match (approximately two hours) and two hours after. The performances of the FTP service for 1 and 10 MB file size, including the end-user throughput and the setup time, are presented in Figure 3.

A clear differentiation can be inferred in each of these time intervals that presents small variations depending on the operator. Additionally, it can be observed from the figures that there were distinct behaviors for the two operators. Notably, for operator A, the phases of the match are easily distinguished, while operator B shows more consistent performance. Notwithstanding, it can be inferred that generally, after the game, the FTP service performs better. This fact might be due to people arriving early to the game and leaving right after the game often. Thus it was observed that the network remained loaded before and during the game.

**Figure 4:**
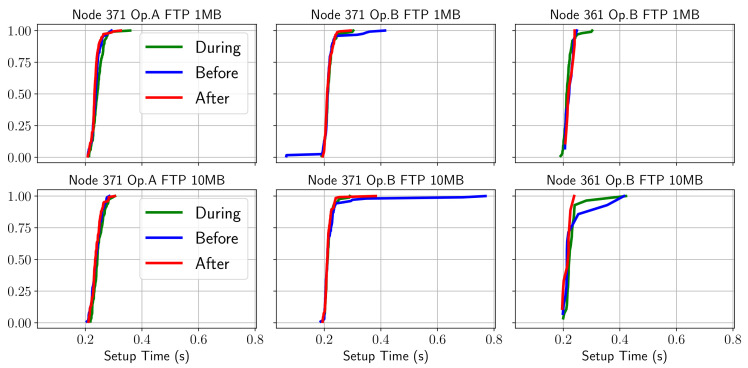
CDF of FTP setup time behavior before/during/after the match.

Regarding the second KQI of the FTP service (i.e., the setup time), the corresponding CDFs are shown in Figure 4, for each node and operator logged in the second campaign. In these, it can be seen that the distinction of the different phases of the match is not clear. However, according to previous studies, this result is expected because it depends more on variables related to the processing capacity than on the state of the radio network.

**Figure 5:**
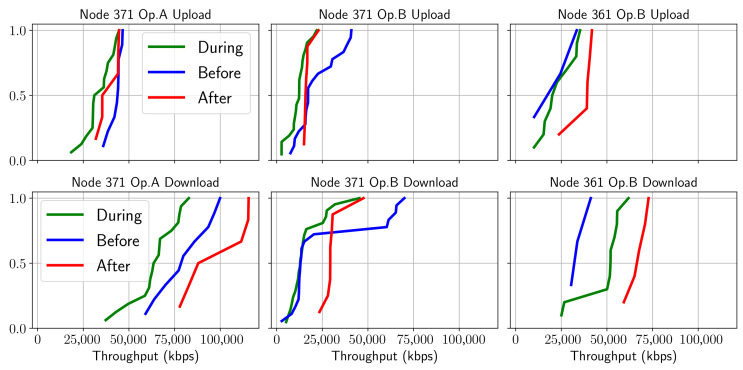
CDF of NetTest throughput behavior before/during/after the match; the upper row corresponds to uploading and lower to downloading.

Concerning the NetTest speed test experiment Figure 5, there was also a clear difference in performance, although in this case, the download and upload patterns were different. Such phenomena can be explained by the types of applications typically employed by users connected to the same base station and their demand for resources. As was the case with FTP, we can observe the pattern of behavior of users loading the cell in terms of download capacity before and during the match with greater intensity than after in almost all cases. That is the case, for example, for the node 371 operator A interface where the performance after the event is better in downloading, unlike uploading where the best result is obtained before the event.

**Figure 6:**
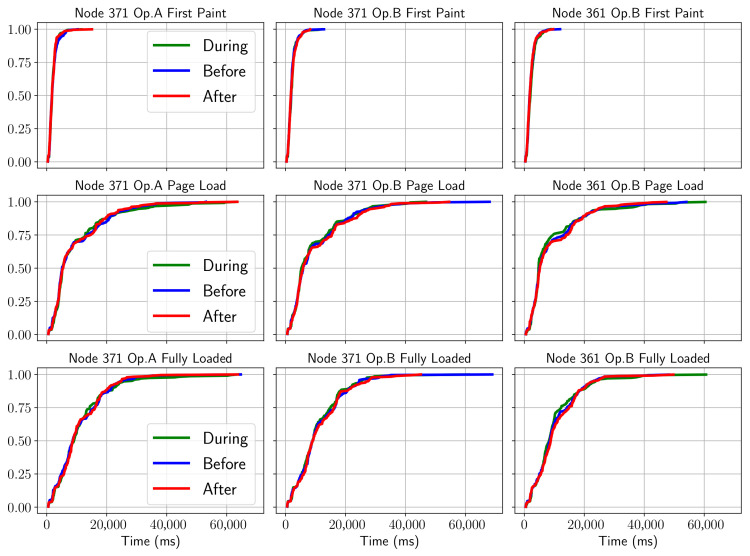
CDF of HTTP KQI behavior before/during/after the match.

In the case of a web service (which includes Flicker, Twitter, Google, Twitter, Etsy, tmall, eBay, Facebook, LinkedIn, Instagram, the guardian, stack overflow, Reddit, Yelp, Wikipedia or Microsoft pages), it can be seen from the CDF representations that it is not possible to clearly distinguish each of the phases shown in Figure 6. In addition, the behavior between operators cannot be characterized either.

**Figure 7:**
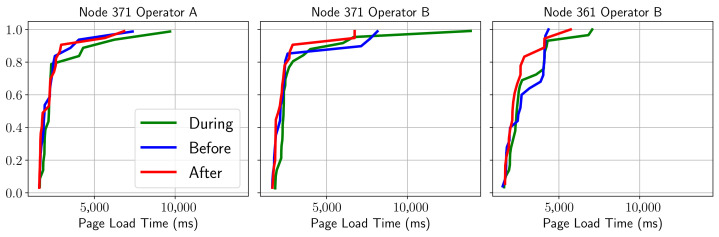
CDF of HTTP page load time (Wikipedia main page) before/during/after the match.

This could be explained in part by the asynchronous nature of the service and considering the performances of different web pages. Thus, in order to have a more accurate view, it is necessary to filter a specific site (i.e., Wikipedia), as shown in the Figure 7. According to this graph, although the separation between the time phases is somewhat better appreciated, it is not possible to determine consistent patterns, with results being different for each node. In this case it is easier to see a stage differentiation, thereby confirming the previous hypothesis about the consistency of the data. It can be established that in order to obtain an adequate pattern it is necessary to test by comparing a single site and if possible obtained from the same server.

**Figure 8:**
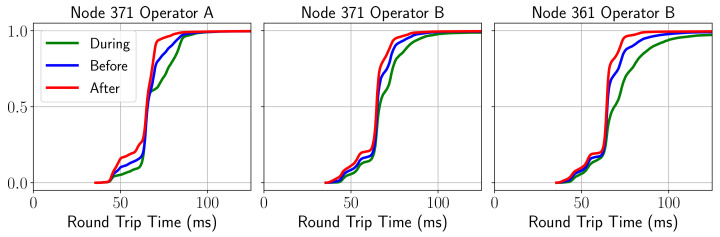
CDF of ping behavior before/during/after the match.

To conclude this section, the results obtained for the KQI of the ping service (i.e., RTT) corroborate the hypothesis that there is different behavior in each of the phases of the game, degrading especially during and before it, and improving afterwards. Figure 8 shows the CDF for each of the phases, and unlike the previous cases, the behaviors of both operators were fairly similar.

As can be deduced from the figure, during the course of the match a higher delay was noticed, probably due to a higher network load. The behavior before and after the match resulted in a lower latency, although in the post-match period the best overall performance in this sense. This common pattern was replicated in the same way for all the services studied regardless of node and operator.

### 4.5. Radio Environment Modem Analysis

Once KQI performance ranges have been identified for various services, it becomes necessary to illustrate the conditions of the radio environment in which they have taken place. For this purpose, a MODEM Log data collection of 10 days without events (Normal day) has been performed to compare with the data collected in campaigns 1 and 2.

**Figure 9:**
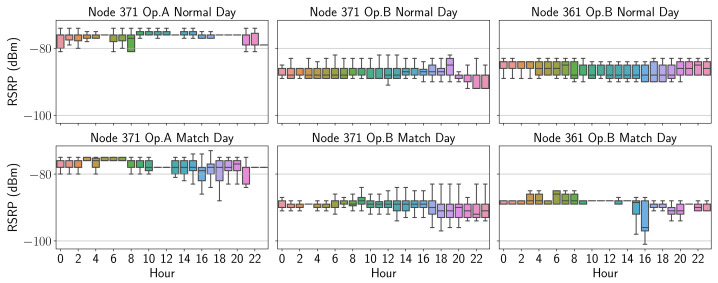
RSRP boxplot comparison.

**Figure 10:**
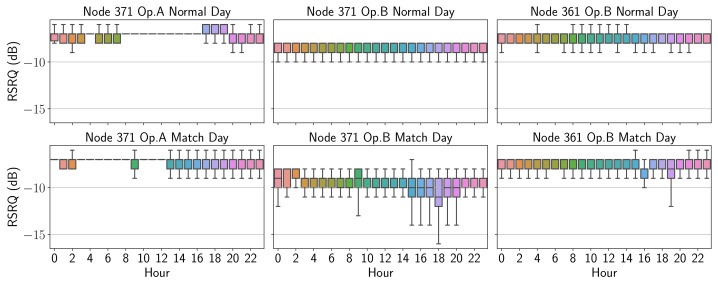
RSRQ boxplot comparison.

Thus, Figure 9 shows in the form of an hourly boxplot the RSRP values over an entire day, the upper part corresponding to a “normal” day and the lower part to the events. Likewise, Figure 10 shows the values corresponding to RSRQ.

It is possible to visualize if there is a consistency across matches compared to the behavior of a “normal” (without event) day. By observing the time series of relevant radio parameters, a clear difference in each operator’s behavior in relation to the course of a match can be noticed. Here the operator A can be seen to drop from an average RSRP of around −80 dBm in normal conditions to values below from 16:00 on match days. On the other hand, the RSRQ values, which for operator B remain above −10 dB in normal conditions, are significantly lower in the afternoon at both nodes. Lastly, it is evident that the graph concerning the parameters obtained on a "normal" day shows a pattern with less variability compared to match days.

### 4.6. Radio Layers’ Packet Granularity

Given the diverse nature of the data sources, a wide range of heterogeneous temporal granularities can be found. Experiments can be performed with a sampling period within the range of minutes. At the same time, the radio interface provides updates with the logs approximately every second. Therefore, the variation over time is an essential consideration for the correlation between samples and overlapping the time series of the different parameters.

**Figure 11:**
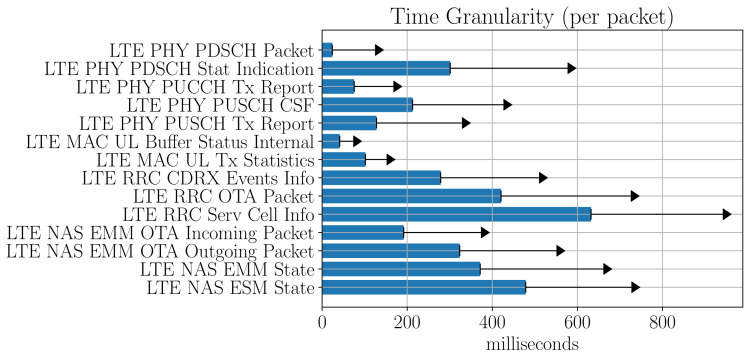
Mobile insight trace packet time granularity.

The Figure 11 shows an example of how the temporal granularity varies in the case of the packets contained within the Mobile Insight traces. The bars show the average time between packets captured, with the arrow indicating the standard deviation.

An apparent disparity between the packets in the case of the physical layer, and for example, those collected in the RRC layer can be appreciated. For the correlation analysis with the NetTest service, the PDSCH LTE Packet’s content has been chosen, since it corresponds to the highest granularity of all the information collected by Mobile Insight traces. It also contains the key PHY layer parameters, such as MCS, transport block size (TBS) and carrier index used (coded as primary or secondary component carrier).

### 4.7. KQI and Radio Logs Correlations

Once the impacts of the development of a social event (in this case a football match) on the QoE and radio behavior patterns of the environment have been observed, the next step is to find out which physical parameters have been relevant for said influence.

**Figure 12:**
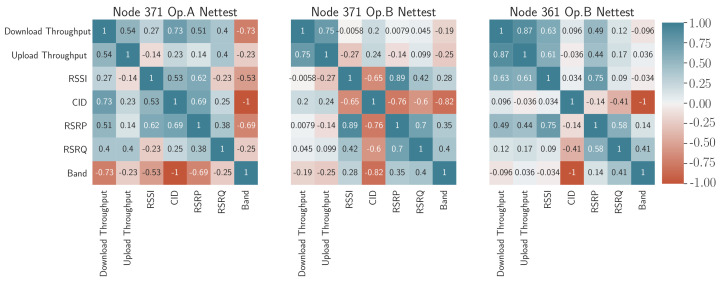
MODEM log parameter–KQI correlations for NetTest.

First of all, a correlation matrix between the parameters obtained in the MODEM log and the KQI corresponding to the NetTest [42] is captured in Figure 12. This matrix shows the correlations between pairs of parameters in a color code that varies in intensity from red (negative correlation with value −1) to dark blue (positive correlation with value 1).

By looking at the radio parameters obtained from the MODEM log, the correlations of these parameters present different fluctuations depending on the operator configuration. This fact is exemplified in 371 Op, a case where it can be deduced that there is a negative correlation between cell identification and upload time, while in the rest of the cases, a higher RSRP value means a better throughput (direct correlation). Based on the results, a higher correlation with CID is shown in node 371 operator B. This fact likely means that the node changes the serving cell with the performance varying depending on its RSRP and RSRQ. Concerning operator A, the RSRP and RSRQ show a relatively appreciable correlation factor for the UL/DL throughput (above 30%). Additionally, noteworthy, although somewhat expected, is the high correlation of the RSSI with the KQI, even higher than that obtained with RSRP, since it is not an averaged value that better reflects the received signal. In this sense, when the selected band has a negative influence on the received signal RSSI (as in node 371, operator A), there is a negative impact on the download throughput (−0.73). For the case of the band for operator B, both node 361 (−0.096) and node 371 (−0.19) present a relatively smoothed influence, even though the latter had its operator A interface in the same device.

Remarkably, both throughputs are closely correlated, especially in operator B (nodes 361 and 371). This fact is related to the effect of the load on the mobile network, which means that when there are many users connected, both upstream and downstream resources are limited at the same time. In other words, when the cell is unloaded, there is a greater probability of finding available resources in both directions.

A further step in exploring the factors most involved in the degradation of NetTest performance indicators is the study of the correlations with some of the physical layer parameters obtained from the Mobile Insight traces. These parameters are the MCS and TBS in layers 0 and 1.

**Figure 13:**
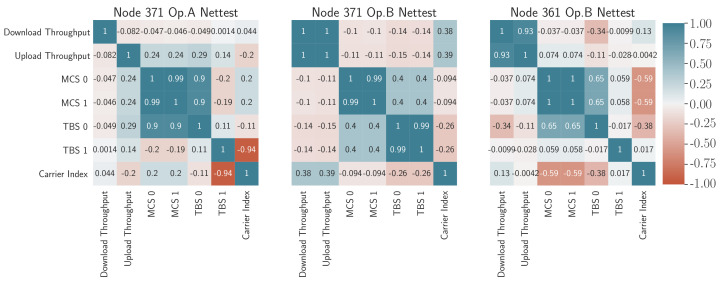
PHY layer parameter–KQI correlations for NetTest.

As shown in the Figure 13, the correlations between KQI and such parameters are not very strong given the low intensity of the correlations (graphically represented with softer colors). This is possibly because there is a more significant interaction with the radio signal conditions that depend on weather directly, and not so much on the performance of the service that is the result of long-term averaging.

In any case, the analysis provided by matrices shown in Figure 12 together with Figure 13 allows identifying patterns of influence between KQIs, and between KQIs and the radio parameters that may be negatively affecting them. That is the case for operator B, where it can be deduced that the change of cell may be the cause of a degraded performance.

## 5. Applications and Research Challenges

**Figure 14:**
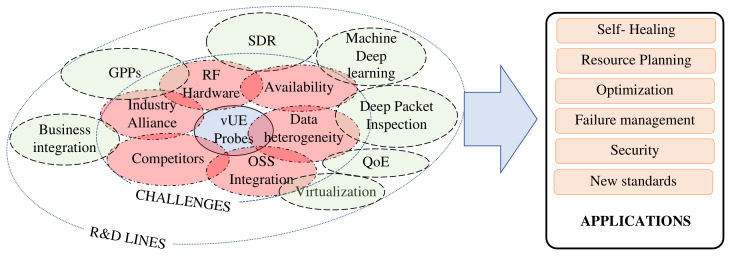
Applications, challenges and identified R&D lines.

The use of the data obtained by the vUE probes either from periodic pre-programming or from direct access through the SSH interface has many different applications, as envisaged in the evaluation section and summarized in Figure 14.

Under the self-healing paradigm, the use of vUE probes can help in detecting problems in the network that are being unnoticed by the OSS system. An example of this is what is known as partial outage or sleeping cells that occur when there is a degradation of a key performance indicator (KPI) without an alarm. The only way for operators to become aware of this type of problem is when the users report low performance. Furthermore, it is necessary to send a qualified technician to the site to determine the cause of the problem. The application of vUE probes would make it possible to detect the degradation process through periodic campaigns of experiments. Additionally, the availability of a temporary database of the user plane performance under normal load conditions represents a valuable input for all the ML algorithms proposed for fault management.

For cell optimization, when operators make changes to the radio network configuration, it is not always feasible for them to assess the consequences. Due to the dynamism of the radio environment and the effects between network layers, it is necessary to have a view from the user end to verify the impact. Additionally, the efficient use of radio resources is another aspect of optimization in which vUEs can play a crucial role. Having a detailed map of the radio environment (with interferers affecting certain areas or parts of the spectrum underutilized in others), such time-stamped information can be leveraged for much more efficient and dynamic management. The heterogeneity of radio technologies that are likely to coexist in the future makes this an essential point to be addressed. Additionally, in relation to physical signal processing, some of the paradigms associated with the simultaneous transmission and reception of signals will have to be tackled in order to design systems capable of managing data of different nature in real time.

Besides, traffic profiling using the most advanced deep learning techniques can serve as input for better resource planning. It can also get a more accurate picture of how many resources are consumed depending on the application the user gives them. Moreover, from a QoE management perspective, having a user interface integrated into the network management system makes it possible to better manage the quality of service without invading data privacy.

The security of mobile networks, including identity management and privacy protection, would benefit significantly from the presence of vUE. Among the many threats that could be detected is the possible presence of rogue stations attempting to impersonate the network. Furthermore, through the use of vUEs, periodic pen-testing could be carried out to check the vulnerability of network access.

In the same manner, the use of the probes could be extended to other use cases still under study by the 5G standardization bodies. For example, the vUE probes could communicate with other user devices that are out of coverage by acting as relays. Thus, some of the experimental features proposed at the forefront of standard development could be piloted to test their operational feasibility in commercial networks. Enabling these tests would result in a shorter time to market that would boost the revenue of operators and foster competition.

However, as identified by the evaluation section, the present implementations of vUE probes present relevant challenges. First of all, the efforts of strategic alliances aimed at joining forces for an open mobile radio technology must converge in a solution of wide dissemination that suits all parties, as happened with the WiFi alliance. To make this a reality, the SDR technologies would have make a to leap in capacity compared to what they offer now, since at the moment, the bands they are capable of covering do not yet include millimeter wave (mmWave) and bandwidths beyond 20 MHz. Therefore, there is still a critical hardware development challenge to be overcome.

Furthermore, even if large pools capable of supporting the instantiation of vUEs are available, their management should be integrated with the actual OSS. Therefore, the heterogeneity present in the ecosystem of network management technologies would be increased along with its complexity. Linked to this challenge is the necessity to consider the products currently performing similar tasks of study of mobile networks through drive tests, which would constitute direct competition for the entry of vUEs into the market. Third parties must enter the business so that this type of service could be outsourced.

A further important consideration is the diverse nature of the information to complete the contextual sources, such as sensors and the easiness of its availability. In this way, the expected increase of computing capabilities in the cloud (or on the cloud edge) could provide resources for the vast amount of information that a massive deployment of vUEs would imply. Therefore, a horizon is opening up in which all the aspects, as mentioned earlier, will gradually come together for an automatic management setup capable of tackling the most challenging problems with a precision never seen before.

Last but not least, there would be a multitude of technical challenges to overcome in order to integrate the output in the form of logs into the big data platforms that feed the machine learning algorithms under development [43]. Among them, the diversity of periodicities of each of the measurements can be highlighted [44] as a field to be explored with the implementation of vUE probes. Besides, and based on the timestamped measurements gathered, specific novel time-series processing techniques would have to be applied [45,46] to obtain greater precision in the analysis and its inclusion in the network management algorithms.

## 6. Conclusions

The present work has presented the primary information sources and key approaches for monitoring in current cellular network OAM, while identifying their shortcomings. As a means to solve them, vUE probes have been demonstrated to be a very relevant tool. From this, an integrated framework for its application has been designed, establishing its capabilities to support advanced monitoring and management tasks.

The different parts that would be necessary for integration with the network management system have been described, along with the mechanisms for their operation and implementation.

Then, a summary of all the efforts made so far to be able to integrate the data obtained from the user in the automatic network management for various purposes has been presented in addition to the works that propose the virtualization of probes in any of its conceptions.

A real-world implementation of this framework has demonstrated the capabilities of the proposed approach for advanced QoE analysis and the identification of the relations between the large number of parameters involved in the service provision, and provided novel insights on how crowd-events can affect the network performance.

In this sense, it has been studied how the occurrence of an event, by its nature, produces a pattern of degradation in specific time periods. It has also been shown how the correlations of the radio parameters with the performance indicators or KQI can vary depending on the configuration or strategy of the operator for users in the same relative positions.

Thus, the benefits of using vUE have been synthesized by taking advantage of the technological momentum of the co-evolution of virtualization and open radio platform technologies, together with the rapid development of new user applications.

The identified open research areas and challenges pave the way for further research and developments in the field to guide future tools for advanced cellular network management.

As future work, a more extensive study of the measurement campaign, including analysis of variance (ANOVA) and the inclusion of the MAC and RRC network layers, would complete the analysis provided here. 

## Figures and Tables

**Table 1:** Key quality indicators of the service tests.

Application	KQIs	Other Parameters
Hyper Text Transfer Protocol (HTTP)	First Paint (ms)	Page Timings Navigation Timings Resource Timings Ping
	Page Load Time (ms)	
	Fully Loaded Time (ms)	
File Transfer Protocol (FTP)	Setup Time (s)	N/A
	Total time (s)	
	Speed Download (Mbps)	
NetTest	DL/UL Throughput (kbps)	N/A
	Resolution Time DL/UL (ns)	
	Total bytes DL/UL	
Ping	RTT (ms)	N/A
Video Streaming	Initial Delay (s)	Highest quality used Most used quality Time spent per quality
	Goodput (Mbps)	
	Number of Stallings	
	Number of up down quality switch	
	Buffer size (bits)	

**Table 3:** Descriptions of measurement campaigns.

	Match	Date	Event Hour	Attendance	MI Samples	MODEM Samples	Ping Samples	Web Samples	NetTest Samples	FTP Samples
Campaign 1	Match 1	16/8/18	20:45	8028.0	0	2194	0	144.0	0	0
	Match 2	26/8/18	18:00	16,519.0	0	2345	0	143.0	0	0
	Match 3	2/9/18	18:00	15,427.0	0	2521	0	144.0	0	0
	Match 4	26/9/18	20:00	6579.0	0	2143	0	136.0	0	0
	Match 5	4/10/18	18:55	11,484.0	0	2006	0	170.0	0	0
	Match 6	1/11/18	19:05	7908.0	0	2380	0	171.0	0	0
	Match 7	4/11/18	18:00	13,983.0	0	2447	0	240.0	0	0
	Match 8	8/11/18	21:00	12,386.0	0	2226	0	175.0	0	0
	Match 9	29/11/18	18:55	14,061.0	0	2276	0	180.0	0	0
	Match 10	5/5/19	20:00	10,040.0	0	2959	0	56.0	0	0
	Match 11	16/5/19	18:00	15,427.0	0	2651	0	118.0	0	0
	Match 12	19/5/19	18:00	13,038.0	0	2728	0	132.0	0	0
Campaign 2	Match 1	14/9/19	18:00	12183.0	71	4356	145	48.0	8.0	182
	Match 2	28/9/19	20:00	12,578.0	32	3286	196	48.0	0	42
	Match 3	3/10/19	21:00	10,296.0	139	2478	107	131	134	118
	Match 4	27/10/19	20:00	14,093.0	150	3894	184	0	162	647
	Match 5	10/11/19	20:00	12,039.0	90	3621	191	0	641	710
	Match 6	1/12/19	18:00	11,026.0	124	2139	192	0	444	190

## Data Availability

Not applicable.

## Abbreviations

The following abbreviations are used in this manuscript:

5G	Fifth Generation of Mobile Networks
ANOVA	Analysis of the Variance
APN	Access Point Name
BBU	Base Band Unit
BS	Base Station
CDF	Cumulative Distribution Function
CID	Cell ID
COTS	Commercial Off-The-Shelf
DNS	Domain Name System
eNodeB	Evolved Node B
FTP	File Transfer Protocol
gNodeB	next Generation Node B
GPP	General Processing Platform
HTTP	Hyper Text Transfer Protocol
ICMP	Internet Control Message Protocol
LTE	Long Term Evolution
KPI	Key Performance Indicator
KQI	Key Quality Indicator
MAC	Media Access Control
MCC	Mobile Country Code
MCS	Modulation and Coding Scheme
MNC	Mobile Network Code
MDT	Minimization of Drive Test
MI	Mobile Insight Trace
ML	Machine Learning
NAS	Network Access Stratum
NET	NETwork Layer
NFV	Network Virtualization Function
NWDAF	Network Data Analytics Function
OAM	Operation, Administration and Maintenance
OSS	Operational Support Systems
PDSCH	Physical Downlink Shared Channel
PHY	Physical Radio Layer
QoE	Quality of Experience
RAN	Radio Access Network
RLC	Radio Link Control
RRC	Radio Resource Control
RSRP	Reference Signal Received Power
RSRQ	Reference Signal Received Quality
RTT	Round Trip Time
RRM	Radio Resource Management
PDCP	Packet Convergence Control Protocol
SDN	Software Defined Network
SDR	Software Defined Radio
SIM	Subscriber Identity Module
SSH	Secure SHell
SQL	Structured Query Language
TBS	Transport Block Size
TCP	Transport Control Protocol
UE	User Equipment
vUE	Virtual UE probe
WiFi	Wireless Fidelity
